# Ponatinib Protects Mice From Lethal Influenza Infection by Suppressing Cytokine Storm

**DOI:** 10.3389/fimmu.2019.01393

**Published:** 2019-06-21

**Authors:** Si Chen, Ge Liu, Jungang Chen, Ao Hu, Li Zhang, Wenyu Sun, Wei Tang, Chunlan Liu, Haiwei Zhang, Chang Ke, Jianguo Wu, Xulin Chen

**Affiliations:** ^1^State Key Laboratory of Virology, Wuhan Institute of Virology, Chinese Academy of Sciences, Wuhan, China; ^2^University of Chinese Academy of Sciences, Beijing, China; ^3^Wuhan Virolead Biopharmaceutical Company, Wuhan, China; ^4^Guangzhou Key Laboratory of Virology, Institute of Medical Microbiology, Jinan University, Guangzhou, China

**Keywords:** cytokine storm, immunomodulator, influenza A virus, ponatinib, pro-inflammatory cytokine

## Abstract

Excessive inflammation associated with the uncontrolled release of pro-inflammatory cytokines is the main cause of death from influenza virus infection. Previous studies have indicated that inhibition of interferon gamma-induced protein 10 (IP-10), interleukin-8 (IL-8), monocyte chemoattractant protein 1 (MCP-1), or their cognate receptors has beneficial effects. Here, by using monocytic U937 cells that capable of secreting the three important cytokines during influenza A virus infection, we measured the inhibitory activities on the production of three cytokines of six anti-inflammatory compounds reported in other models of inflammation. We found that ponatinib had a highly inhibitory effect on the production of all three cytokines. We tested ponatinib in a mouse influenza model to assess its therapeutic effects with different doses and administration times and found that the delayed administration of ponatinib was protective against lethal influenza A virus infection without reducing virus titers. Therefore, we suggest that ponatinib may serve as a new immunomodulator in the treatment of influenza.

## Introduction

Influenza has attracted extensive attention due to its remarkably high mortality and morbidity with 650,000 deaths worldwide associated with seasonal influenza-caused respiratory diseases (1). Antiviral drugs that inhibit influenza virus replication have been previously approved to treat influenza, but have significant drawbacks, including drug resistance and short therapeutic windows that limit their clinical applications (2, 3). In addition, a major cause of patient death from influenza is acute respiratory distress syndrome, which is caused by excessive inflammation in the lung (4). Therefore, therapies that suppress exaggerated inflammatory responses and immune-mediated pulmonary injury could also be effective at reducing the mortality and morbidity from influenza. Currently, there have been no effective immunomodulatory agents available for treating severe influenza, because classical anti-inflammatory drugs, such as corticosteroids, have been reported to worsen patient outcomes in the clinic. Therefore, novel drugs aiming to suppress the inflammation induced by influenza are urgently needed (5, 6).

Levels of multiple pro-inflammatory cytokines, such as interleukins (ILs), tumor necrosis factor (TNF), interferons (IFNs), and chemokines, in the samples of patients with severe influenza, increased. The robust production of inflammatory cytokines is often called a “cytokine storm” or “cytokine dysregulation” (7). Previous studies have indicated interleukin 6 (IL-6), IL-8, monocyte chemoattractant protein 1 (MCP-1), interferon gamma-induced protein 10 (IP-10), tumor necrosis factor-α (TNF-α), and C-C motif chemokine ligand 5 (CCL-5) are highly expressed in the lung tissues from fatal human cases and animal models (8, 9). Mice with a genetic deletion on the gene of one of these cytokines or their cognate receptors were generated to verify the roles of these cytokines in the observed inflammation and to find suitable therapeutic targets. Only deficiencies in MCP-1 receptor (CCR2) or IP-10 were protective during influenza infection (10, 11). Mice with other cytokine genes knocked out, such as TNF-α, IL-6, or CCL-5 receptor (CCR5), exhibited only modest improvements in morbidity and delays in mortality (12, 13). Meanwhile, a few drugs targeting cytokines have been reported to be protective against influenza. IP-10 antibody and IL-8 receptor (CXCR1/2) antagonist are among those observed to be protective (11, 14). It appears that the inhibition of a single cytokine is insufficient to ameliorate the pathology following influenza virus infection. For example, a multi-cytokine inhibitor, such as the sphingosine-1-phosphate receptor agonist AAL-R, is protective against influenza, suggesting global suppression of inflammatory factors may be a better strategy of treating influenza (15).

Unfortunately, very few multi-cytokine inhibitors have been developed, likely due to the complexity of the cellular signaling pathways involved in cytokine storms. However, inhibitors of many signaling pathways have been developed in recent years, with a great number of compounds being approved for antitumor therapy. Many studies have indicated that multiple signaling pathways involved in tumors are also essential for cytokine production (16). Small molecule inhibitors of inflammation associated signaling pathways have been used for repressing cytokine release in inflammation induced by lipopolysaccharide, bleomycin, etc. While there are limited reports on inhibitors with anti-inflammatory effects on influenza therapy. Based on the previous study showing inhibition of IP-10, MCP-1, and IL-8 or their cognate receptors was beneficial in the treatment of influenza, a monocyte cell line U937 capable of secreting IP-10, MCP-1, and IL-8 during influenza virus infection was used to identify inhibitors against cytokine responses induced by influenza virus infection from six potent compounds, all of which have not been reported in anti-influenza field but were demonstrated to have therapeutic effects in other inflammation models. We found ponatinib has the highest activity to reduce the levels of all three pro-inflammatory cytokines in human U937 cells. Furthermore, the therapeutic effect of ponatinib was confirmed in mouse influenza model. Ponatinib reduced significantly the mortality in mice caused by H1N1 influenza A virus infection accompanied by reduced expressions of multiple cytokines and inflammatory injury in the lungs. Here, we propose ponatinib should be considered for further preclinical development for the treatment of influenza as an immunomodulatory agent.

## Materials and Methods

### Cell Lines, Animals, and Virus Strains

Madin-Darby canine kidney (MDCK) cells (ATCC CCL-34) were cultured in Dulbecco's modified Eagle's medium. Human pulmonary epithelial (A549) cells (ATCC CCL-185) and human monocytic cell line U937 (ATCC CRL-1593.2) were maintained in RPMI-1640 medium. All media was supplemented with 10% fetal bovine serum, 100 U/ml penicillin, and 100 U/ml streptomycin. All these cell types were maintained at 37°C in a 5% CO_2_ incubator.

Influenza virus strain A/PuertoRico/8/1934 (H1N1) was provided by the virus collection at the Wuhan Institute of Virology of the Chinese Academy of Sciences in China. Virus stocks were prepared using 10-day-old embryonated chicken eggs. The virus titers were measured using hemagglutination and 50% tissue culture infective dose (TCID_50_) assays in MDCK cells.

BALB/c mice (6–8 week) were purchased from the Beijing Vital River Laboratory Animal Technology Co., Ltd (Charles River laboratories China) and housed under specific pathogen-free conditions. All experiments were conducted according to the protocol approved by the Animal Care and Use Committee of Wuhan Institute of Virology of the Chinese Academy of Sciences (WIVA08201201).

### Chemicals

Sorafenib, nilotinib, PP2, BMS-345541, TWS119, and ponatinib were purchased from MedChemExpress Co., Ltd (Shanghai, China). All test compounds were initially dissolved in DMSO.

### Cytokine Assay

The concentrations of cytokines were measured by Alphalisa assay. Briefly, 20 μl of acceptor beads and 5 μl of supernatant were added to each well of 384-well OptiPlates, which were then incubated in the dark at room temperature for 1 h. Next, 25 μl of donor beads coated with streptavidin, which captures the biotinylated antibody, was added. After the assay, plates were incubated in the dark at room temperature for 0.5 h, the assay plates were read in AlphaScreen mode on an Envision plate reader (Wallac Envision, PerkinElmer, MA, USA).

### Cytotoxicity Assay

Compound toxicity was determined by CellTiter-Glo Assay (Invitrogen). Briefly, after adding 15 μl of CellTiter-Glo reagent, plates were incubated at room temperature for 15 min with shaking. The luminescence intensity was determined by a multi-label plate reader (Wallac Envision, PerkinElmer, MA, USA).

### Western Blot Assay

Cells were homogenized in RIPA lysis buffer containing 1% protease inhibitor cocktail (Roche) to extract total protein. Equal amounts of protein homogenates were separated by SDS-PAGE (Bio-Rad) and transferred onto polyvinylidene difluoride membranes (pore size 0.45 μM, Bio-Rad), which were then blotted with monoclonal antibodies against GAPDH (1:1000, ZSGB-BIO), p38, phospho-p38, STAT1, and phospho-STAT1 (1:1000, Cell Signaling Technology). Proteins were detected with corresponding horseradish peroxidase-tagged secondary antibodies and enhanced chemiluminescence Western blot reagents (Advansta) and visualized using an enhanced chemiluminescence system (AlphaEase FluorChem System, Alpha Innotech Corp.).

### Animal Experiment

Female BALB/c mice (6–8weeks old) were anesthetized by intraperitoneal injection of sodium pentobarbital (75 mg/kg) and then intranasally infected with the mouse-adapted influenza A/PR8 virus (500 TCID_50_, dissolved in PBS) in a volume of 20 μl. The mock group was inoculated with virus diluent. Ponatinib dissolved in 25 mM citrate buffer (pH 2.75) in a volume of 200 μl was administered orally. The placebo group was administered orally with drug diluent. Following infection, mice were observed daily for signs of disease, such as lethargy, ruffled hair, and weight loss. Once mice succumbed to infection, their body weight would not be counted. On day 2, 3, 5, and 7 post-infection, the mock and infected mice were sacrificed. The tracheas and lungs were removed and washed three times by injection of 2 ml of PBS containing 0.1% BSA. After centrifugation at 3,000 rpm, the bronchoalveolar lavage fluids (BALFs) was collected. Concentrations of IFN-α and IFN-β were measured by ELISA (PBL Biomedical Laboratories) according to the manufacturer's instructions. Concentrations of IL-1β, IL-2, IL-3, IL-4, IL-5, IL-6, IL-9, IL-10, IL-12(p40), IL-12(p70), IL-13, IL-17A, Eotaxin, G-CSF, GM-CSF, IFN-γ, Keratinocyte Chemoattractant (KC), MCP-1, MIP-1α, MIP-1β, RANTES, and TNF-α were assessed using a Bio-Plex Pro Mouse Cytokine Grp I Panel 23-plex (Bio-Rad) according to the manufacturer's instructions and read on a Bio-Plex MAGPIX System (Bio-Rad). Concentrations of IP-10 were measured by Alphalisa assay (PerkinElmer).

### Flow Cytometry

BALF samples were washed with FACS buffer (10% BSA in PBS). Red blood cells were lysed using ammonium chloride and 10 cells/well were seeded into a 96-well U-bottom plate. Cells were pre-incubated with anti-FcgRIII/II (Fc block) in FACS buffer before a 30-min incubation with the following fluorochrome-labeled antibodies (Biolegend): PE-Cy7-conjugated Ly6C (clone HK1.4, dilution 1:200), PE-conjugated CD11b (clone M1/70, dilution 1:200), APC-conjugated Ly6G (clone 1A8, dilution 1:200), and FITC-conjugated CD11c (clone N418, dilution 1:100). Cells were washed with phosphate-buffered saline twice and counterstained with 7AAD (Biolegend) to differentiate apoptotic and dead cells and then analyzed using a LSR Fortessa (Becton Dickinson).

### Histology

Whole lungs were perfused with 10% neutral buffered formaldehyde *in situ*. Tissues were then fixed overnight in 10% neutral buffered formaldehyde, embedded in paraffin, and sectioned. Lung specimens were stained with hematoxylin and eosin and then subjected to gross and microscopic pathologic analysis.

### Preparation of Murine Precision-Cut Lung Slices (PCLS)

PCLS were prepared using a modification of a protocol that has been previously reported (17). After anesthetization by intraperitoneal injection of sodium pentobarbital (75 mg/kg), the mouse was bled through the abdominal aorta. Then, the trachea was exposed, dissected from surrounding tissues and was cannulated with an 18-gauge needle. Through the cannula, the lung was inflated with 1.3 ml of 2% low-melting agarose (BIO-RAD) dissolved in Hank's buffered saline solution (HBSS) solution. The whole lung was cooled with ice for 10 min to solidify the agarose. Then, the lung was taken from the thoracic cavity and placed in the slice culture medium (Dulbecco's Modified Eagle Medium: Nutrient Mixture F-12, DMEM/F-12, GIBCO) at 4°C for an additional 15 min to completely solidify the agarose. The culture medium was supplemented with 100 units/ml of penicillin, 100 μg/ml streptomycin, and 250 ng/ml of amphotericin to avoid contamination. The lung lobe was afterward dissected and cut to create a flat surface at the end of the primary bronchus. Another flat surface was cut ~0.8 cm from the first surface. The cube was maintained in the pre-chilled slice culture medium prior to or during the slicing. The cube was cut into slices of desired thickness using a vibratome slicer (Leica, VT1200S). Each mouse lung cube generated at least 250 μm slices. The slices were then transferred into a 48-well cell culture plate and covered with 250 μl of slice culture medium in each well. The medium was changed every hour at least three times before virus infection to remove cell debris.

### Statistical Analyses

The concentrations required to inhibit cytokine production by 50% (EC_50_), reduce cell viability by 50% (CC_50_) and selective indices (SIs, which is equal to CC_50_/EC_50_) of compounds were calculated using Prism v.5 software (GraphPad Software, San Diego, CA). Data were presented as mean ± SD for each point. Differences between averages between control samples and tests were statistically analyzed using Student's *t*-test, *p*-values <0.05 were considered statistically significant. For body weight studies, two-way repeated measures ANOVA with *post-hoc* Bonferroni *t*-test was performed. Survival of mice was compared using the Log-Rank (Mantel-Cox) test.

## Results

### Ponatinib Was Identified to Inhibit the Production of Three Important Cytokines Associated With Influenza-Induced Cytokine Storm

**Figure 1:**
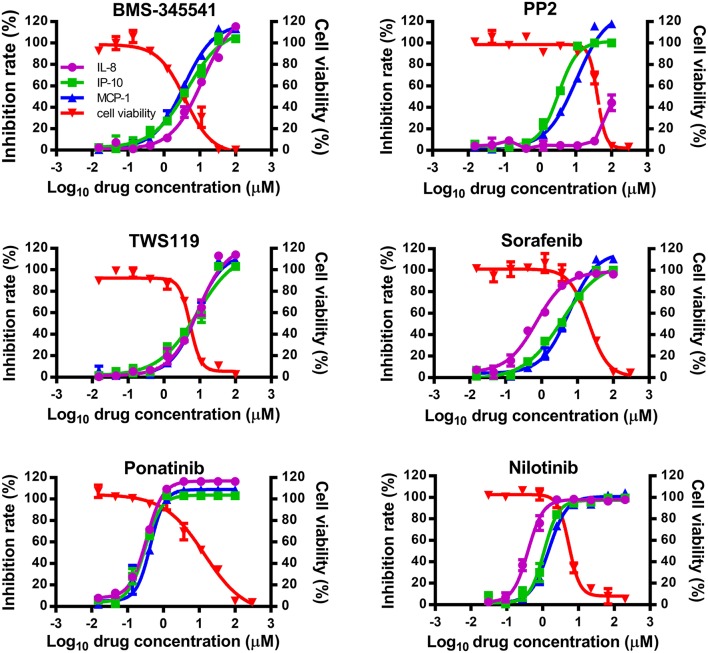
Ponatinib was identified to inhibit the production of three important cytokines associated with influenza-induced cytokine storm. U937 cells were infected with influenza A virus A/PuertoRico/8/1934 (MOI = 0.1) in the presence or absence of serially diluted of each compound and incubated at 37°C for 48 h. The supernatants were harvested, and concentrations of IL-8, IP-10, and MCP-1 were measured by AlphaLISA. Cell viability was also determined using Cell Titer-Glo. The kinetic curves of each compound were made using Prism v.5 software (GraphPad Software, San Diego, CA). The values represent the means ± S.D. of duplicate samples from three independent experiments.

To study whether inhibitors of cytokines and chemokines that contribute to influenza-induced inflammation are effective to reduce the inflammatory response in influenza virus-infected cells, six small-molecule inhibitors were selected to evaluate their inhibitory activity in human U937 cells infected with influenza virus. Among these inhibitors, BMS-345541 can suppress 17 cytokines (such as IL-6, IL-8, IP-10) secretion in human fetal lung fibroblasts infected by human parainfluenza viruses (PIV) (18). PP2 can reduce macrophage inflammatory protein-2 (MIP-2), MCP-1, MIP-1α, and CCL-5 levels in the inflammation model of acute-pancreatitis-like changes (19). TWS119 can inhibit MCP-1 production induced by TNF-α in spinal astrocytes (20). Sorafenib, nilotinib, and ponatinib were reported to reduce chemotherapeutic drugs induced IL-1β, IL-6, and the chemokine (C-X-C motif) ligand 1 (CXCL1) in bone marrow-derived macrophages (21). Nilotinib was also reported to reduce IL-6, IL-1β, and TNF-α levels in mice of bleomycin-induced acute lung injury model (22). As shown in Figure 1, among the 6 inhibitors tested, only ponatinib showed significant inhibitory effects on the production of all three cytokines, with SIs of 46.3, 46.3, and 34.8 for IL-8, IP-10, and MCP-1, respectively. PP2, nilotinib, and sorafenib were found to have high inhibitory effects on single cytokine production. PP2 inhibited IP-10 production with an SI of 12.2, while sorafenib and nilotinib inhibited IL-8 production with SIs of 29.6 and 13.5, respectively. The rest of the inhibitors did not show any inhibitory activity (SI < 10). Overall, ponatinib was identified to have inhibitory activity on the production of IL-8, IP-10, and MCP-1 in U937 cells infected by H1N1 influenza virus PR8 strain (Figure 1, Table 1).

**Table 1:** Selected compounds for anti-inflammatory test.

**Name**	**CC_**50**_**	**EC**_****50****_			**SI**			**Target**
		**IL-8**	**IP-10**	**MCP-1**	**IL-8**	**IP-10**	**MCP-1**	
BMS-345541	3.9	11.7	4.6	3.6	0.3	0.8	1.1	IκB/IKK
PP2	40.3	>100	3.3	10.4	<0.4	12.2	3.9	Src
TWS119	5.4	9.0	8.5	8.0	0.6	0.6	0.7	GSK-3
Sorafenib	20.7	0.7	3.7	5.8	29.6	5.6	3.6	Raf
Ponatinib	13.9	0.3	0.3	0.4	46.3	46.3	34.8	Bcr-Abl, VEGFR, FGFR, PDGFR, Flt
Nilotinib	5.4	0.4	1.1	1.5	13.5	4.9	3.6	Bcr-Abl

### Ponatinib Reduces the Mortality in Mice Infected With Lethal Influenza A Virus

**Figure 2:**
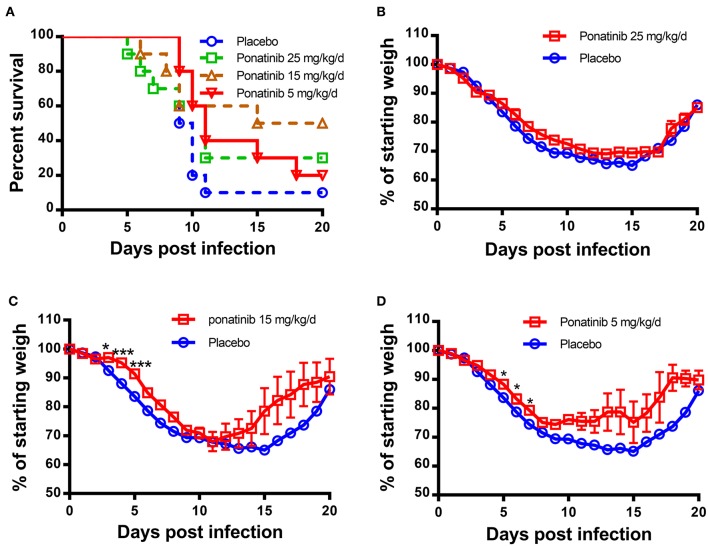
Ponatinib reduces influenza A virus-induced mortality in mice. BALB/c mice of 6–8 weeks old were infected with 500 TCID_50_ of influenza A virus (A/PR/8/34) by intranasally. Two hours later, 4 groups of mice (*n* = 10) were simultaneously treated orally with 25, 15, 5 mg/kg/d of ponatinib, or placebo. Survival rate **(A)** and body weight loss **(B–D)** were monitored daily until day 20 post-infection. The data are representative of at least three independent experiments.*p < 0.05; ***p <0.001.

Since ponatinib was identified to inhibit the production of three important pro-inflammatory cytokines in human monocyte U937 cells, it was tested for a therapeutic effect on mouse influenza model infected by H1N1 influenza PR8 virus. Twenty-five mg/kg/d of ponatinib was set as the maximal drug dose administered in PR8-infected mice (23). As shown in Figure 2A, the placebo-treated mice started dying from day 9 and by day 11 and 90% of them had succumbed to infection. The mice treated with 15 mg/kg/d of ponatinib showed the highest survivalrate (50%) and had the least decline in body weight during the early stage (days 3 to 5) of influenza A virus infection (Figures 2A,C). The mice treated with 5 mg/kg/d of ponatinib showed lowest survive rate (20%), and the improvement in body weight loss decreased significantly compared to the middle dose group (Figures 2A,D). However, mice in the high dose group (25 mg/kg/d) also showed low survive rate (30%), and improvement of body weight loss was not observed at all (Figures 2A,B). Taken together, the therapeutic effect of ponatinib on mouse influenza model was confirmed, and 15 mg/kg/d was chosen as the optimal dose for further *in vivo* study.

### Delayed Administration of Ponatinib Enhances Protection Against Lethal Influenza Virus Infection in Mice

**Figure 3:**
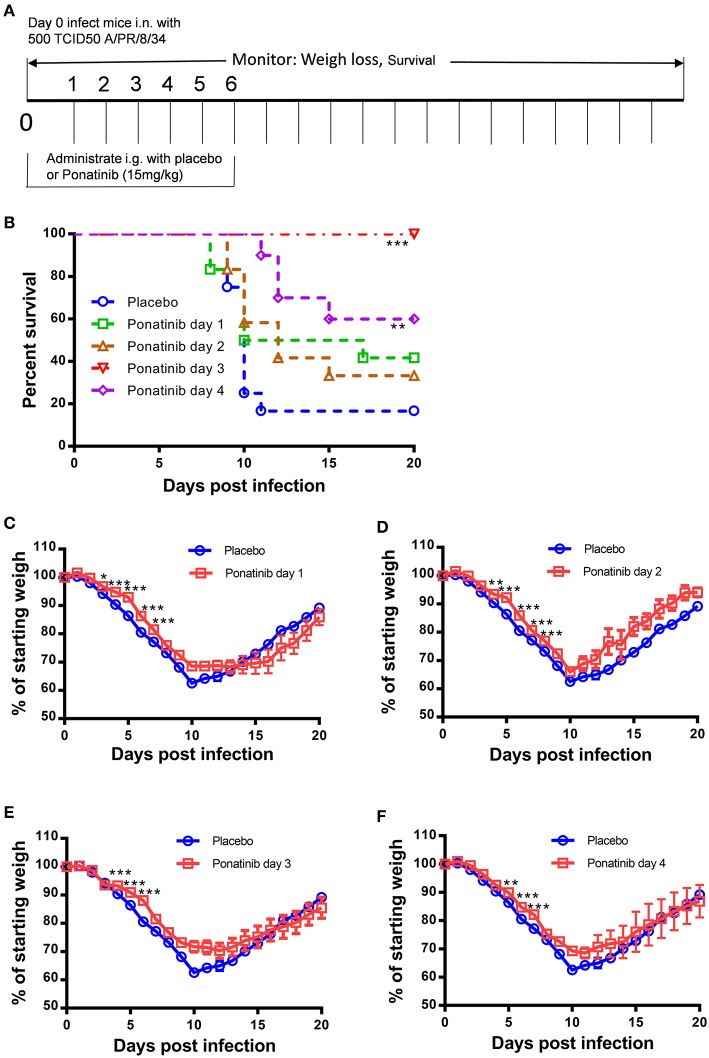
Delayed administration of ponatinib enhances protection against lethal infection in mice. **(A)** Experimental setup for optimization of drug administration timing. Mice were infected as described in Figure 2 but treated with ponatinib (15 mg/kg) starting on days 1, 2, 3, or 4 post-infection until day 6 post-infection. Survival rate **(B)** and weight loss **(C–F)** were monitored daily until day 20 post-infection. The data are representative of at least three independent experiments. *p < 0.05; **p < 0.01; ***p <0.001.

To explore the optimal time to start ponatinib treatment, we performed the *in vivo* experiments with drug administration started on days 1, 2, 3, or 4 post-infection (Figure 3A). The mice treated with ponatinib starting on days 3 and 4 had higher survival rates than those treated starting on days 1 and 2 (Figure 3B). The body weight loss of the mice slowed down significantly after the delayed administration of ponatinib (Figures 3C–F). Unlike current antivirals that need to be administered early after virus infection, ponatinib works better when administered starting at days 3 and 4 post-infection when mice have developed obvious clinical symptoms, including piloerection, hunched posture, reduced movement, and labored breathing concomitant with a significant decrease in body weight.

### Ponatinib Suppresses Neutrophils Infiltration in the Lungs of Mice With Lethal Influenza Virus Infection

**Figure 4:**
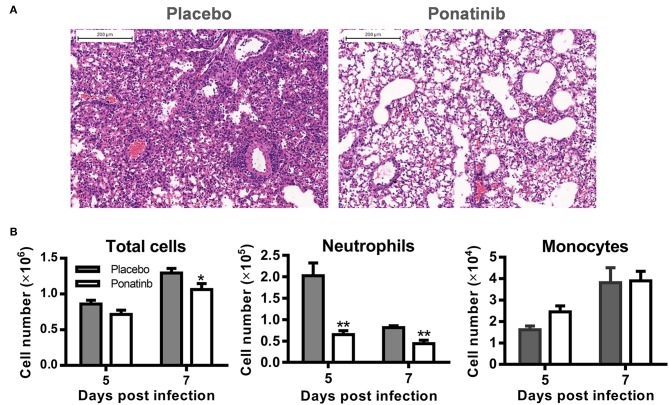
Ponatinib suppresses neutrophils infiltration in the lungs of mice with lethal infection. Mice were infected as described in Figure 2. Starting with day 3, daily administration of placebo or ponatinib (15 mg/kg) was given orally**. (A)** Hematoxylin & eosin-stained mouse lung sections (scale bar 200 μm) harvested at day 7 post-infection. **(B)** BALFs were collected from placebo and ponatinib-treated mice (*n* = 3 per group at each time point) starting day 3 post-infection to monitor total cell counts (mean ± SEM) on days 5 and 7 post-infection. The data are representative of at least three independent experiments.*p < 0.05; **p < 0.01.

To verify the anti-inflammatory activity of ponatinib on the infiltration of inflammatory cells, ponatinib-treated (treatment starting at day 3 post-infection) and placebo-treated mice were euthanized 7 days post-infection to obtain lung tissues for histopathologic examination. In mice treated with the placebo, extensive lung damage, including apoptosis or necrosis of degenerating cells and extensive cellular infiltrates, was observed. There were fewer inflammatory infiltrates observed in the lungs in ponatinib-treated mice than in the lungs of mice treated with placebo (Figure 4A). The cell infiltrates in the BALFs of mice treated with ponatinib or placebo were statistically analyzed for cell numbers and types (Figure 4B). Ponatinib greatly reduced the infiltration of neutrophils, which have been proven to contribute to acute lung injury in influenza pneumonia, while monocyte infiltration was not affected (24).

### Ponatinib Attenuates Pro-inflammatory Cytokines in the Lungs of Mice With Lethal Influenza Infection

**Figure 5:**
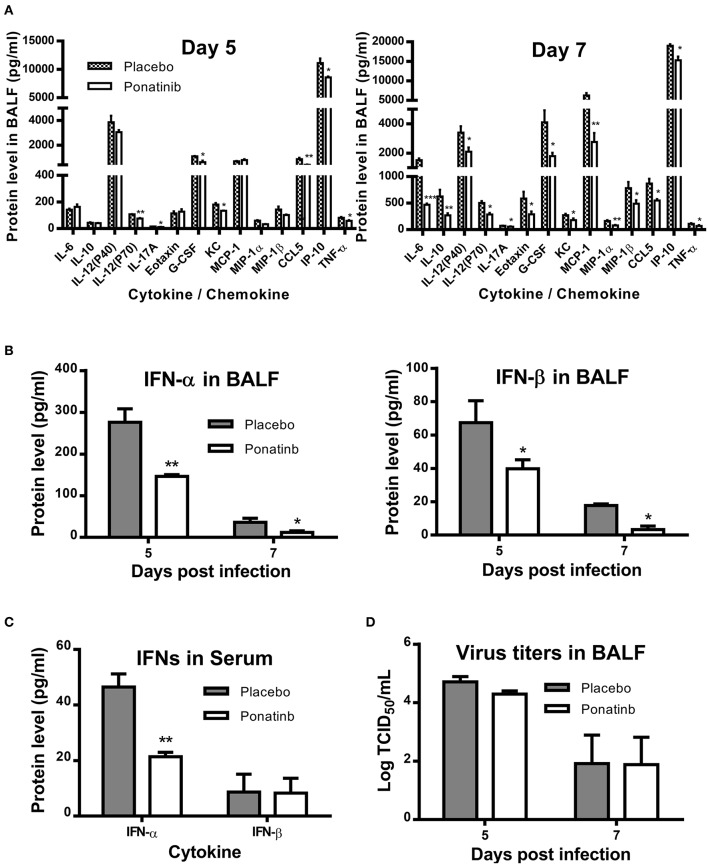
Ponatinib attenuates pro-inflammatory cytokines in the lungs of mice with lethal infection. Mice were infected as described in Figure 2. Starting with day 3, daily administration of placebo or ponatinib (15 mg/kg) was given orally. Inflammatory cytokine/chemokine concentrations in BALF from mice at day 5 and day 7 post-infection (*n* = 3 per group at each time point) were measured. **(A)** IL-6, IL-10, IL-12(P40), IL-12(P70), IL-17A, Eotaxin, G-CSF, KC, MCP-1, MIP-1a, MIP-1β, TNF-α, and CCL5 concentrations were quantified by multiplex assay and IP-10 concentrations were measured by AlphaLISA. IFN levels in **(B)** BALF and **(C)** serum were measured by ELISA. **(D)** Viral titers in BALF taken on day 5 and 7 post-infection were measured by TCID50 assay using MDCK cells (*n* = 3 per group at each time point). The data are representative of two independent experiments.*p < 0.05; **p < 0.01; ***p < 0.001.

We measured the protein levels of 24 cytokines and chemokines in the BALF samples and found that the concentrations of 17 cytokines and chemokines increased significantly during influenza infection. Of these, expression levels of 14 cytokines and chemokines were attenuated at day 7 post-infection in the ponatinib-treated group (Figure 5A) and there were no differences in expression in the remaining 3 cytokines (data not shown). Production of 8 cytokines and chemokines was reduced in mice upon ponatinib treatment at day 5 post-infection, whereas the concentrations of IL-6, IL-10, MCP-1, Eotaxin, IL-12 (p40), and MIP-1β were not affected (Figure 5A). Type I IFNs were also measured and ponatinib was found to reduce the levels of type I IFNs in both BALF and serum (Figures 5B,C). We determined the virus titers in the BALF samples of mice in the placebo and ponatinib groups and found no difference between these groups at days 5 and 7 post-infection (Figure 5D). In the *in vitro* antiviral assays performed using U937, A549 cells, and murine PCLS, ponatinib did not inhibit the replication of the PR8 strain of influenza virus (Figure S1). Therefore, ponatinib does not have any antiviral activity during treatment *in vitro* or *in vivo*. Taken together, our results suggest the anti-inflammatory activity of ponatinib is the main contributor to its protection of mice from lethal influenza virus infection.

### Simultaneous Administration of Ponatinib and Infection of Mice Promotes Viral Proliferation

**Figure 6:**
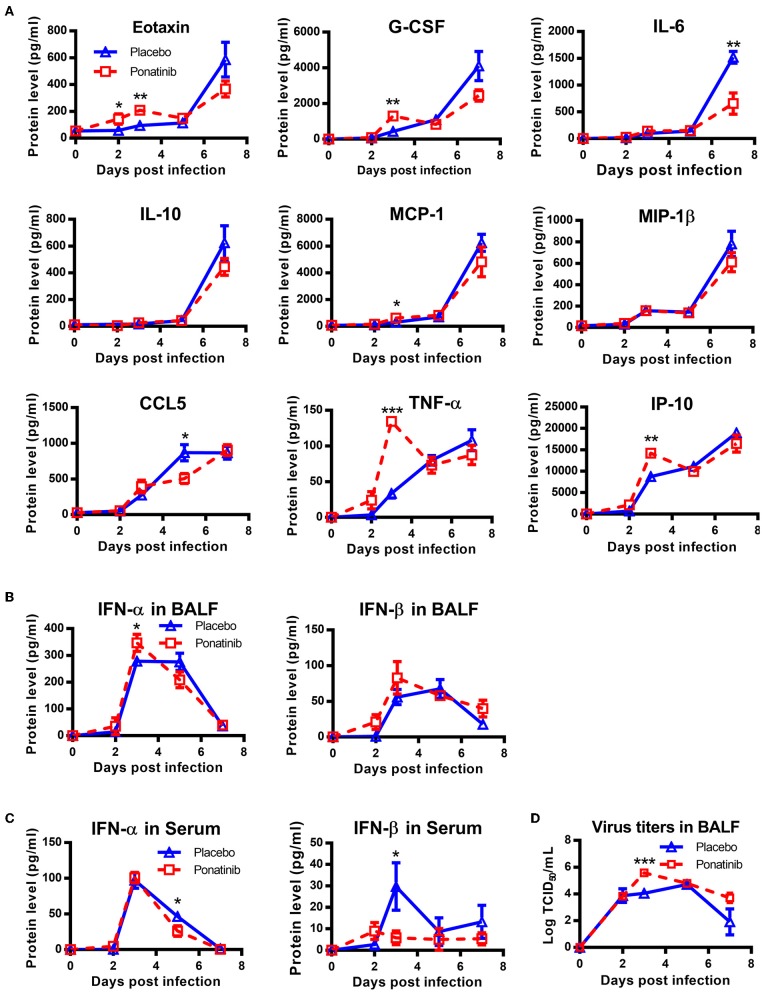
Simultaneous administration of ponatinib and infection of mice promotes viral proliferation. Mice were infected as described in Figure 2. Starting with day 0, daily administration of placebo or ponatinib (15 mg/kg) was given orally. Inflammatory cytokine/chemokine concentrations in BALF from mice at day 0, 2, 3, 5, and 7 post-infection were measured. **(A)** IL-6, IL-10, Eotaxin, G-CSF, MCP-1, MIP-1β, TNF-α, and CCL5 concentrations were quantified by multiplex assay and IP-10 concentrations were measured by AlphaLISA. IFN levels in BALF **(B)** and serum **(C)** were measured by ELISA. (**D)** Viral titers in BALF collected at day 0, 2, 3, 5, and 7 post-infection were measured by TCID50 assay using MDCK cells (*n* = 3). The data are representative of two independent experiments. *p < 0.05; **p < 0.01; ***p < 0.001.

To understand why early treatment was only slightly protective, the BALFs of placebo- and ponatinib-treated mice at days 2, 3, 5, and 7 post-infection were analyzed using a multi-cytokine chip. In the late phases between 5 and 7 days post-infection, the inhibition of cytokines, except for IL-6 and CCL-5, was minor. Meanwhile, no cytokines were inhibited in the ponatinib group in the early phase between days 2 and 3 post-infection. Furthermore, the levels of some cytokines, such as Eotaxin, G-CSF, MCP-1, IL-10, TNF-α, and IP-10, were elevated in the ponatinib group (Figure 6A). The levels of type I IFNs were also determined, and IFN-α levels in the BALFs of mice in the ponatinib group were higher on day 3 and lower on day 5 post-infection (Figures 6B,C). We tested the viral titers in the BALFs samples and found viral titers were significantly higher at day 3 and slightly higher at day 7 post-infection in mice treated with ponatinib simultaneously with infection than treated with placebo (Figure 6D). Therefore, early administration of ponatinib did not reduce cytokine levels during the early phase of influenza virus infection, but rather promoted viral proliferation and delayed viral clearance. This may contribute to the reduced therapeutic effect of ponatinib as influenza treatments when administrated too early.

## Discussion

Monocytes and their macrophage and dendritic-cell progeny are major producers of cytokines during influenza virus infection. It has been reported that human primary monocytes and derived macrophages and dendritic cells can be infected with influenza viruses and release large amounts of cytokines (25–27). In this study, we picked 6 compounds that have been reported to be effective in other inflammation models with reductionsin cytokines that are important for influenza-induced inflammation and measured their anti-inflammatory activities using the monocytic cell line U937, which can be differentiated into either macrophages or dendritic cells *in vitro* (28, 29). Several studies indicated that alveolar macrophages secreted the similar pro-inflammatory cytokines to that secreted in U937 cells upon influenza virus infection, although there are some differences in life span between alveolar macrophages and monocytes (30–32). We found that ponatinib can inhibit the production of IL-8, IP-10, and MCP-1, among the most critical cytokines in influenza pathogenesis. Considering that other cell types also contribute to the production of cytokines and chemokines during influenza virus infection, we evaluated the anti-inflammatory activity of ponatinib in PCLS, which contain bronchial and alveolar epithelial cells, endothelial cells and alveolar macrophages. We found that ponatinib can also reduce MCP-1, KC (functional analogs of human IL-8), IP-10, and IL-6 productions dose-dependently in PCLS infected with influenza (Figure S4). Since many cytokines and chemokines contribute to influenza-induced “cytokine storms”, our results also showed that ponatinib inhibits the production of most of the cytokines/chemokines assessed *in vivo*.

Ponatinib was reported to repress multiple cytokines production induced by chemotherapeutic drugs such as doxorubicin through the p38 pathway (33). The p38 was also considered as an important regulator in the inflammation induced by influenza virus because p38 knock-out mice showed higher survival rate and milder pathological reaction compared to wild-type mice during lethal influenza infection (34). We measured the phosphorylation of p38 in U937 cells and found that the phosphorylation of p38 was upregulated upon influenza A virus infection and downregulated by ponatinib treatment (Figure S2). Although p38 was not the originally designed target of ponatinib, the off-target effect can be explained by two possible mechanisms. One is ponatinib inhibits ZAK (sterile alpha motif and leucine zipper-containing kinase), an upstream signal molecular of p38 (29). The other is that a conserved Asp-Phe-Gly (DFG) motif was observed in the ATP-binding cleft of p38α, which was determined to be targeted by ponatinib (35).

Ponatinib is a third-generation ABL inhibitor used for the treatment of chronic myelogenous leukemia (CML). ABL is a non-receptor protein kinase involved in cell differentiation, proliferation, division, adhesion, apoptosis, and stress responses (36, 37). The kinase activity of ABL protein is autoinhibited by its SH3 domain (38). A mutation in the ABL gene caused by chromosome translocation within the breakpoint cluster region (BCR) leads to deletion of its regulatory domain. The resulting fusion gene, called BCR-ABL, encodes an unregulated highly active kinase that is associated with CML (39). Therefore, BCR-ABL is a good therapeutic target for CML against which various inhibitors, such as imatinib, nilotinib, bosutinib, and ponatinib, have been developed. ABL protein was also reported to participate in innate immunity through regulating the production of type I IFNs by physical association with mitochondrial antiviral signaling protein (MAVS). The ABL inhibitor STI571 (imatinib) reduces vesicular stomatitis virus (VSV)-induced IFN-β levels in MCF-7 cells (40). Our research showed the ABL inhibitor ponatinib can also downregulate levels of IFN-α in influenza virus-infected U937 cells (Figure S3). *In vivo*, type I IFN signaling may play dual roles in viral pathogenesis and clearance. Although IFNAR-KO mice exhibited increased mortality and morbidity after infection with influenza virus due to significant increases in viral titers, treatment with IFN-α increased mortality and morbidity of influenza-infected mice due to more severe inflammation (41, 42). Based on our results, we suggest reducing type I IFN levels in the late stage of influenza infection may be beneficial. ABL can also activate JAK-STAT signaling, further increasing the production of downstream cytokines, such as IP-10 (43). Our results show ponatinib reduces phosphorylation of STAT1 and production of IP-10 induced by influenza virus infection (Figure S2).

Analysis of the expression of multiple cytokines in influenza virus-infected mice revealed massive increases in the amounts of multiple cytokines, including Eotaxin, G-CSF, MCP-1, IL-10, IL-6, MIP-1β, and IP-10, from 5 to 7 days post-infection, although the levels of these cytokines were much lower at day 3 post-infection (Figure 6A). Meanwhile, the concentrations of type I IFNs peaked at day 3 post-infection in the BALF and serum (Figures 6B,C). These observations have been reported in other influenza models, reinforcing the idea that early chemokines induce recruitment of innate immune cells into the lung that can then release more cytokines, which exacerbates cytokine storm and leads to further damage in the lung (42). Therefore, the progression of influenza infection can be divided into various stages and different types of drugs should be administrated during different stages to yield better therapeutic effects. For example, eritoran, a TLR4 antagonist, is protective when administered starting on 2 days post-infection, but only has limited effects when given 3 h prior to infection (44, 45). Similar results were also observed in our study. These observations suggest inhibition of inflammation caused by influenza can only be achieved during the right therapeutic window. According to our results, the best time to start administration of ponatinib is 3 days post-infection when mice show obvious physical changes due to diseases, such as weight loss and piloerection. Early administration of ponatinib increases viral proliferation and influenza severity, which corroborates with the fact that inflammatory responses are essential for the clearance of the virus, but excessive activation of inflammatory responses causes lung damage and death (46).

We found that ponatinib ameliorated lung pathology accompanied by reduced recruitment of neutrophils in mice infected with influenza. Neutrophils play a dual role during influenza infection. They release cytotoxic substances such as oxygen free radicals and proteases which not only kill the virus but also injure alveolar epithelial cells and capillary endothelial cells. Excessive recruitment neutrophils will lead to the increased permeability of the alveolar-capillary barrier, through which edema fluid infiltrates the air space and cause pulmonary edema (47). There is a study reported that attenuation of the neutrophil response by using a neutrophil-depleting antibody correlated with improved survival following infection of mice with influenza virus (48). However, another study reported that complete depletion of neutrophils using antibody methods decreased control of virus replication in the infected mice, which increased morbidity and mortality (49). Thus, the number of neutrophils should be controlled in the appropriate range to keep the balance which ensures the elimination of pathogens without excessive damage to the body. In our study, we found a reduced number of neutrophils through ponatinib treatment promoted virus replication in the early stage of influenza infection, which became more obvious in mice treated with a higher dose of ponatinib (Figures S5, S6). Therefore, delayed administration of ponatinib still exerts an anti-inflammatory effect without affecting the virus replication, because the early innate antiviral response has been activated.

Ponatinib has been used in the clinic for CML therapy and its adverse effects have been thoroughly investigated. It got a boxed warning alert of the risk of vascular occlusion events, heart failure and liver toxicity (50). However, these adverse events often occurred several months later in the long-term treatments. For example, the median time to initial onset of arterial occlusive events was 13.4 months (51). While anti-influenza therapies are short-course, which minimizes adverse effects. According to the data of clinical trials, the early adverse events of ponatinib such as pancreatitis (median time to first onset, 14 days) were mostly reversible (52).

In this study, we found that ponatinib can repress multi-cytokine production in a monocyte model and suggested ponatinib can reduce cytokine release by inhibiting the JAK-STAT and p38 pathways. We also found ponatinib can ameliorate excessive influenza-induced inflammation by suppressing cytokine storms *in vivo*. Therefore, ponatinib has potential as an immunomodulator for the treatment of severe influenza.

## Data Availability

The raw data supporting the conclusions of this manuscript will be made available by the authors, without undue reservation, to any qualified researcher.

## Ethics Statement

All experiments were conducted according to the protocol approved by the Animal Care and Use Committee of Wuhan Institute of Virology of the Chinese Academy of Sciences (WIVA08201201).

## Author Contributions

SC, GL, JC, JW, and XC contributed to the design of experiments. SC, GL, JC, AH, LZ, WS, WT, CL, HZ, and CK contributed to the conduction of experiments. SC, GL, WT, CL, and HZ contributed to the reagents. SC, JW, and XC contributed to the writing and editing the paper.

### Conflict of Interest Statement

The authors declare that the research was conducted in the absence of any commercial or financial relationships that could be construed as a potential conflict of interest.
