# Supplementary figures and images for: Ponatinib Protects Mice From Lethal Influenza Infection by Suppressing Cytokine Storm
Source: Front Immunol. 2019 Jun 21;10:1393. doi: 10.3389/fimmu.2019.01393 (PMC6598400; doi:10.3389/fimmu.2019.01393)

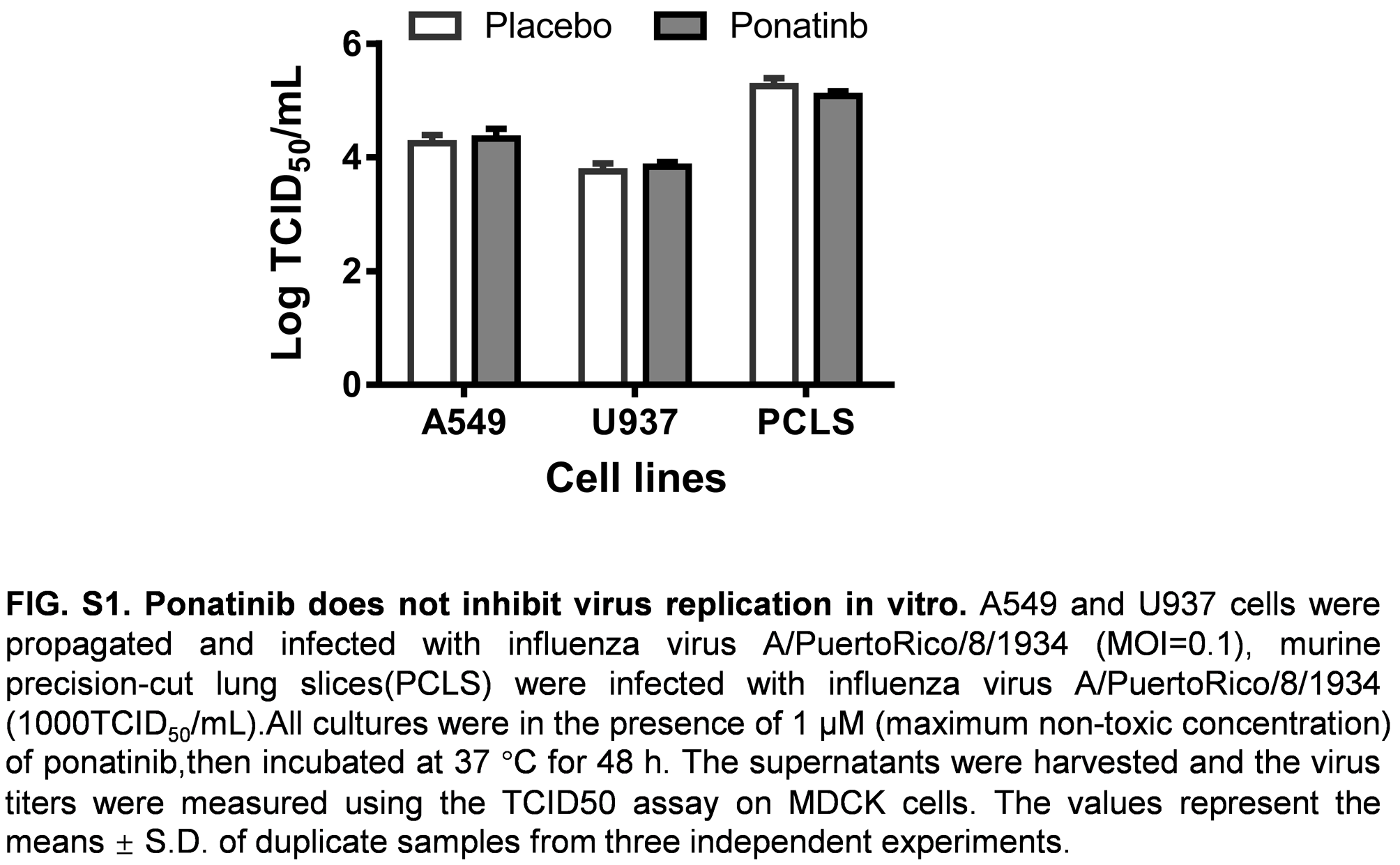

Supplement: Supplementary file 1 [file Image_1.TIF]

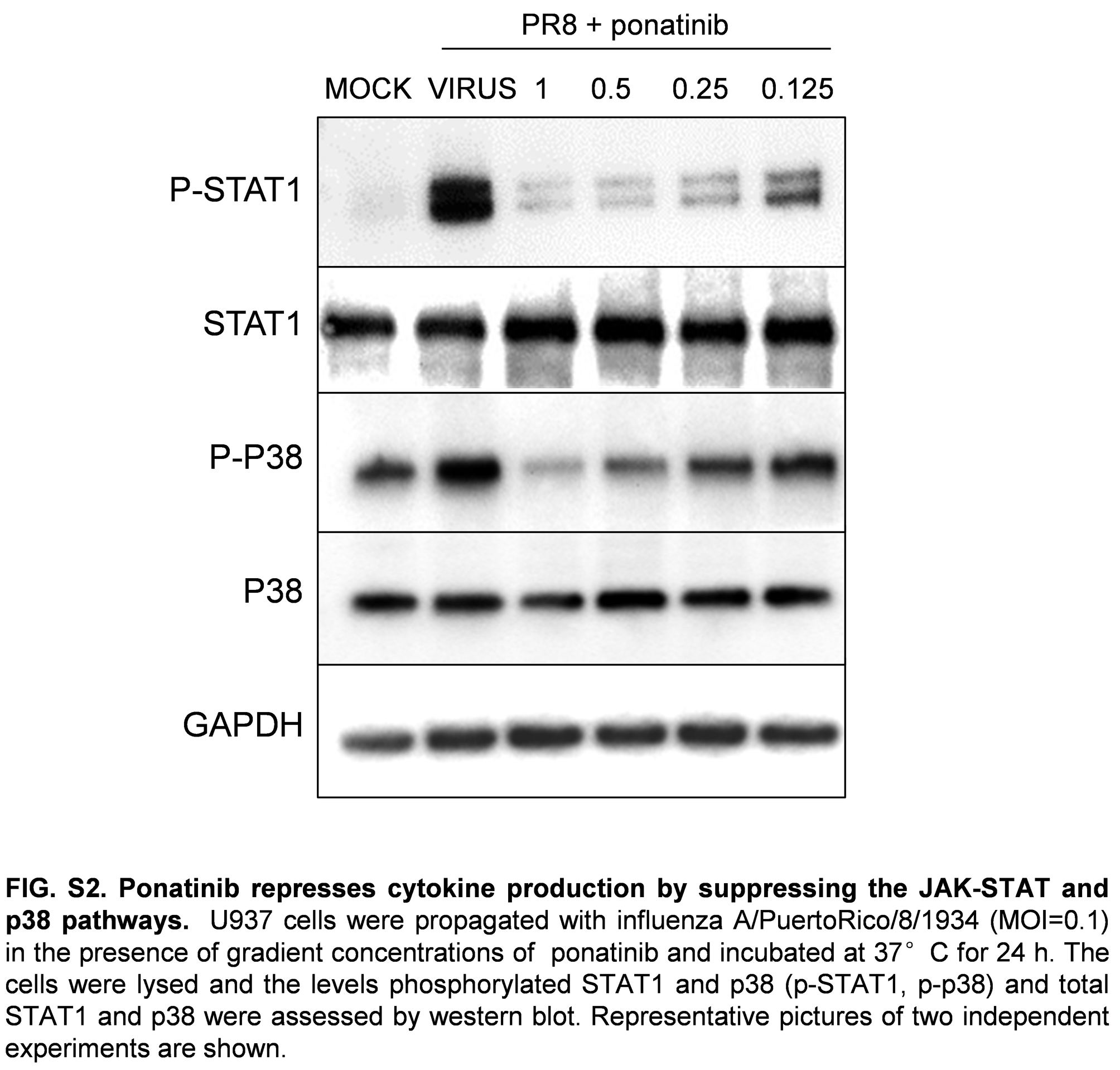

Supplement: Supplementary file 2 [file Image_2.TIF]

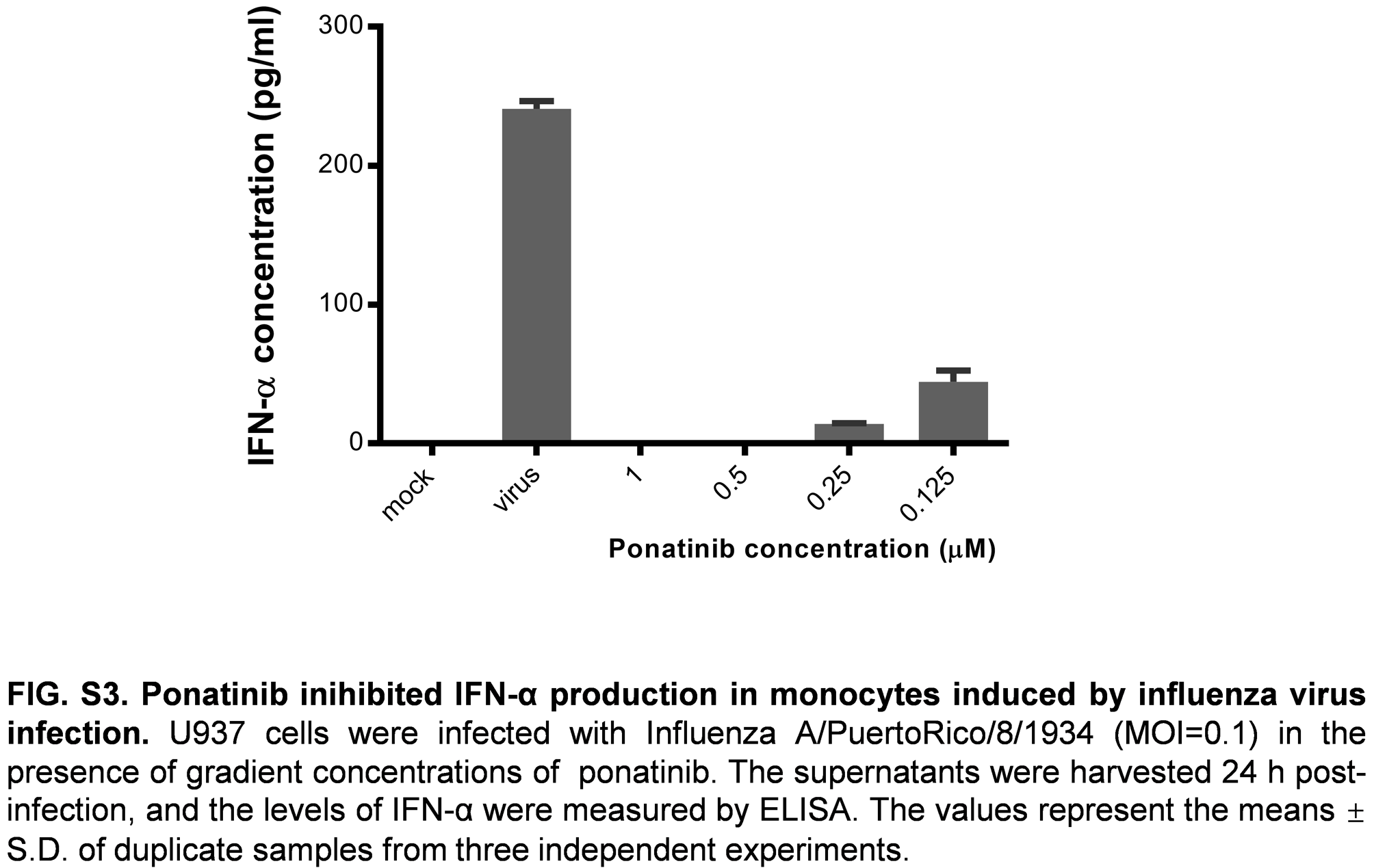

Supplement: Supplementary file 3 [file Image_3.TIF]

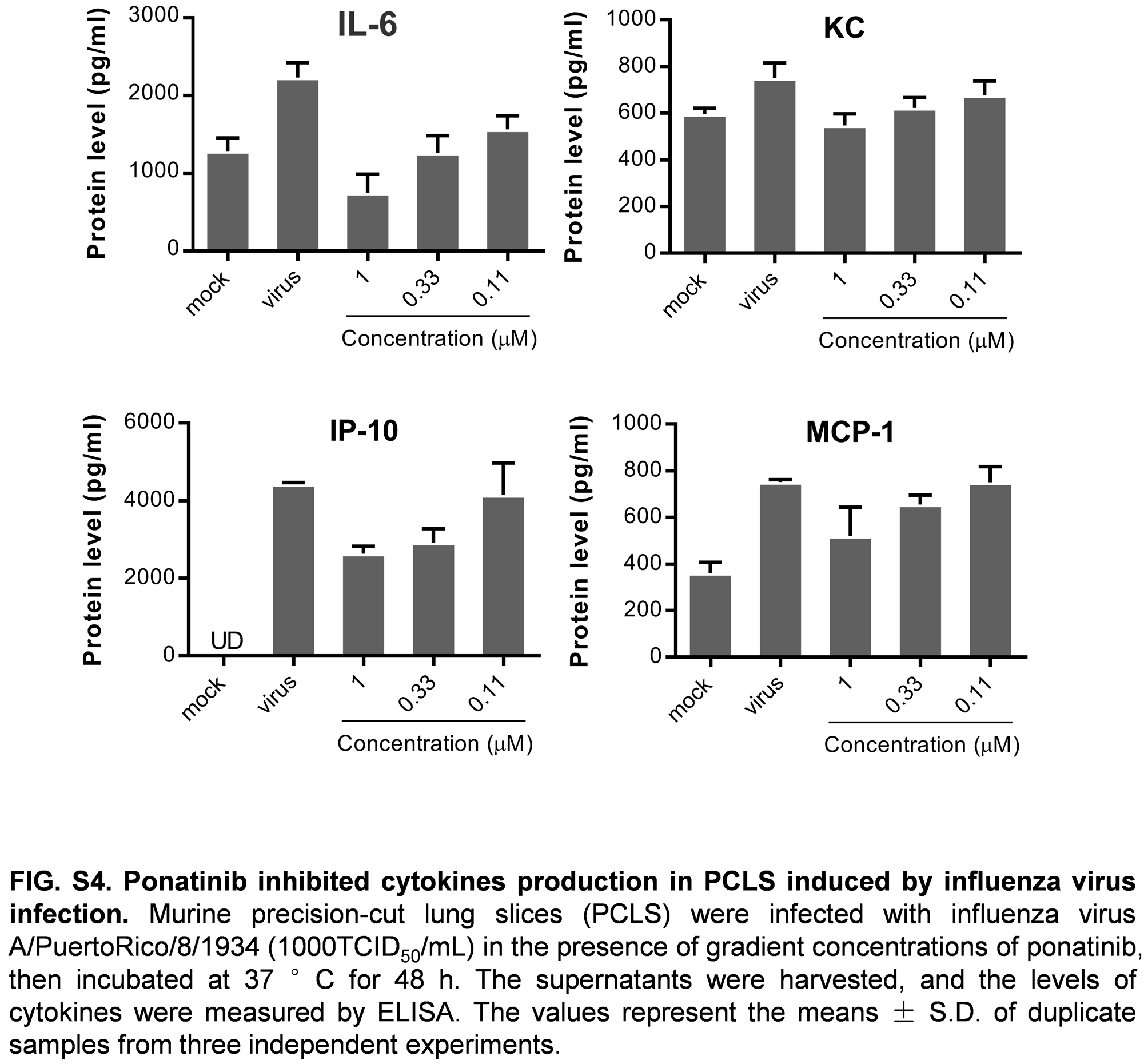

Supplement: Supplementary file 4 [file Image_4.TIF]

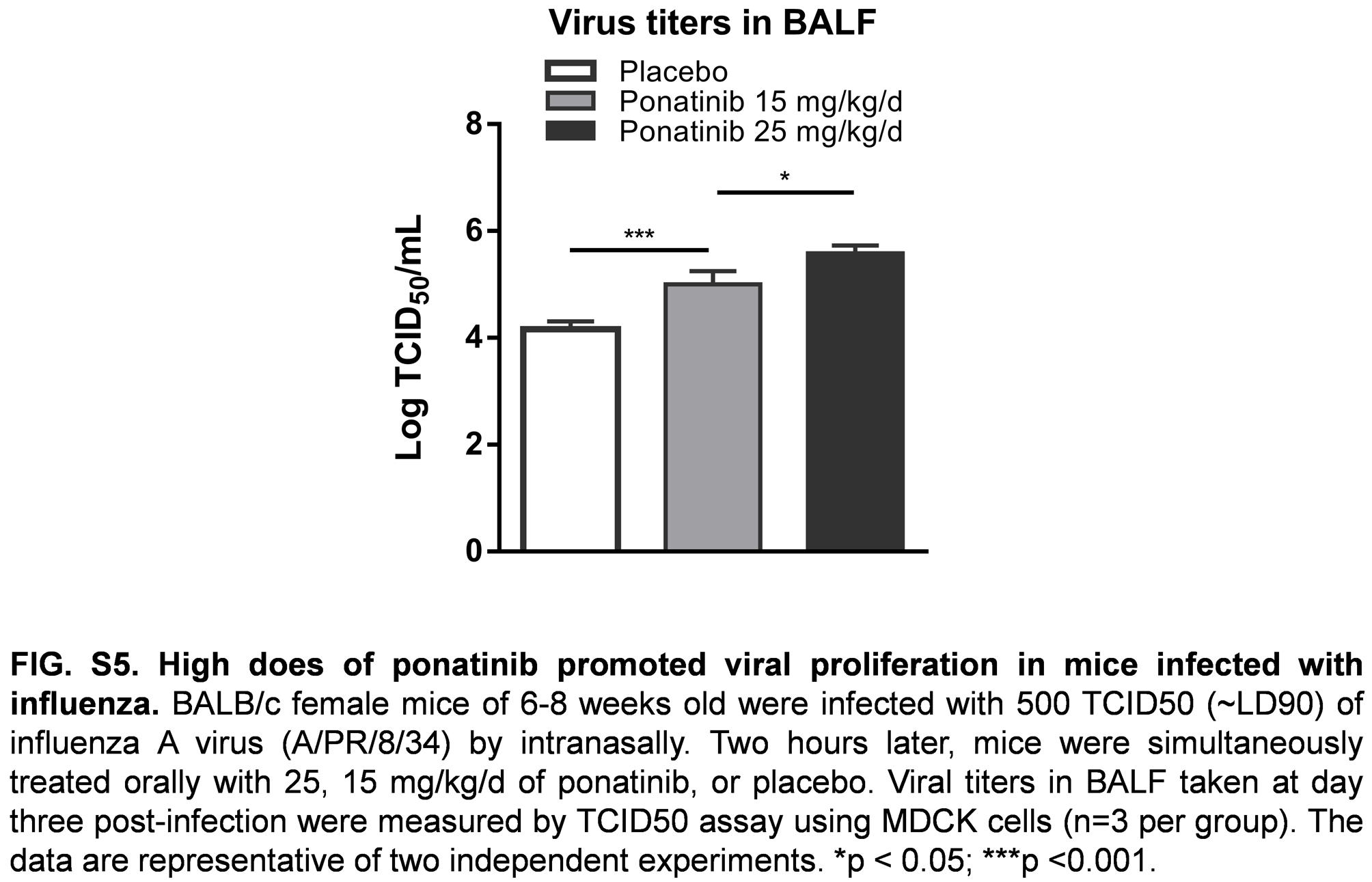

Supplement: Supplementary file 5 [file Image_5.TIF]

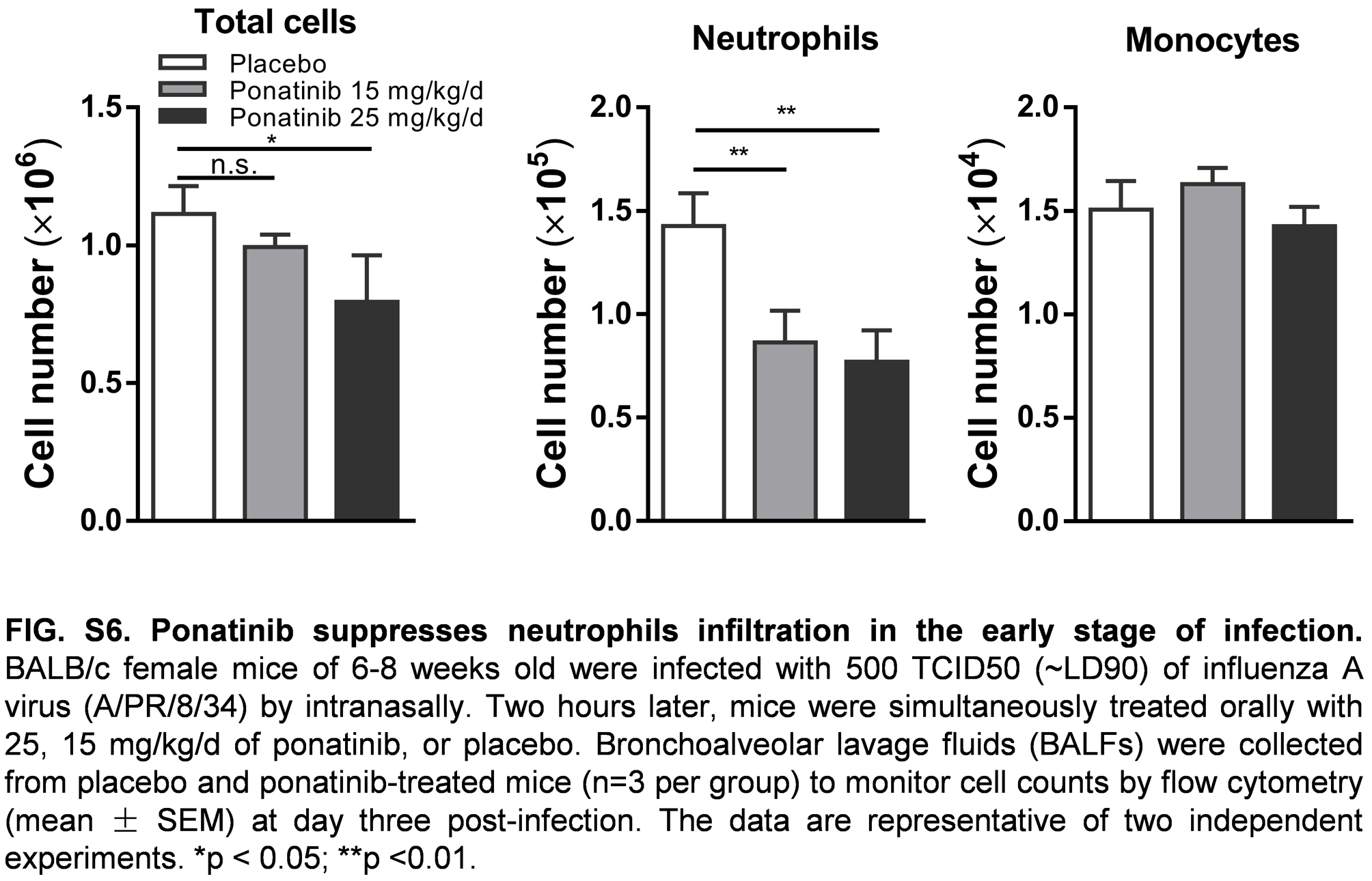

Supplement: Supplementary file 6 [file Image_6.TIF]
